# Brain Imaging Changes in Patients Recovered From COVID-19: A Narrative Review

**DOI:** 10.3389/fnins.2022.855868

**Published:** 2022-04-22

**Authors:** Yan Huang, Qiong Ling, Anne Manyande, Duozhi Wu, Boqi Xiang

**Affiliations:** ^1^Department of Interventional Therapy, The First Affiliated Hospital of Dalian Medical University, Dalian, China; ^2^Department of Anesthesiology, The Second Affiliated Hospital of Guangzhou University of Chinese Medicine, Guangzhou, China; ^3^School of Human and Social Sciences, University of West London, London, United Kingdom; ^4^Department of Anesthesiology, Hainan general Hospital, Haikou, China; ^5^School of Public Health, Rutgers University, New Brunswick, NJ, United States

**Keywords:** SARS-CoV-2, COVID-19, COVID-19 variants of concern, magnetic resonance imaging, brain imaging

## Abstract

The severe acute respiratory syndrome coronavirus-2 (SARS-CoV-2) has caused several outbreaks of highly contagious respiratory diseases worldwide. The respiratory symptoms of Coronavirus Disease-19 (COVID-19) have been closely monitored and studied, while the central nervous system (CNS) and peripheral system (PNS) lesions induced by COVID-19 have not received much attention. Currently, patients with COVID-19-associated encephalopathy present with dizziness, headache, anxiety and depression, stroke, epileptic seizures, the Guillain-Barre syndrome (GBS), and demyelinating disease. The exact pathologic basis for these neurological symptoms is currently not known. Rapid mutation of the SARS-CoV-2 genome leads to the appearance of SARS-CoV-2 variants of concern (VOCs), which have higher infectivity and virulence. Therefore, this narrative review will focus on the imaging assessment of COVID-19 and its VOC. There has been an increase in technologies, such as [^18^F]fluorodeoxyglucose positron emission tomography (^18^F-FDG-PET) and functional magnetic resonance imaging (fMRI), that have been used to observe changes in brain microstructure over time in patients with COVID-19 recovery. Medical imaging and pathological approaches aimed at exploring the associations between COVID-19 and its VOC, with cranial nerve and abnormal nerve discharge will shed light on the rehabilitation process of brain microstructural changes related to SARS-CoV-2, and aid future research in our understanding of the treatment and prognosis of COVID-19 encephalopathy.

## Introduction

### Epidemiology

In December 2019, a severe epidemic respiratory syndrome was reported in Wuhan, Hubei Province, China and was later named coronavirus (2019-nCoV (Xiang et al., 2020; Zhou et al., 2020; Feng et al., 2021a,b, c; He et al., 2021). Coronavirus Disease-19 (COVID-19) is a devastating respiratory disease caused by severe acute respiratory syndrome coronavirus-2 (SARS-CoV-2) (Wu et al., 2020). In October 2021, the COVID-19 pandemic exceeded 240 million confirmed cases around the world and the death toll surpassed 49 million. The number of infections continued to increase so far (Yildiz-Yesiloglu and Ankerst, 2006).

A growing number of case reports and series describe respiratory viruses that can invade the central nervous system (CNS), such as the influenza virus (IV), enterovirus D68 (EV-D68), the human respiratory syncytial virus (HRSV), the coronavirus (CoV), and the human metapneumovirus (HMPV) (Bohmwald et al., 2018; Sooksawasdi Na Ayudhya et al., 2021). The most common manifestation of COVID-19 is acute respiratory distress syndrome (ARDS), in which mild and moderate patients develop headache, cough, ageusia, and anosmia; critically ill patients have acute brain damage after acute respiratory failure, stroke, and metabolic dysfunction; and patients present with persistent altered consciousness and multiple forms of delirium after discharge from hospital (Huang et al., 2020; Lechien et al., 2020; Mao et al., 2020; Pezzini and Padovani, 2020). As the symptoms and long-term effects of the acute COVID-19 become more severe, WHO has developed a clear definition of post-COVID-19. Post-COVID-19 condition occurs in individuals with a history of probable or confirmed SARS-CoV-2 infection, usually 3 months from the onset of COVID-19 with symptoms that last for at least 2 months and cannot be explained by an alternative diagnosis (Soriano et al., 2022). There was evidence of acute cerebrovascular events in 62% of patients and persistent psychiatric disorders, such as delirium and seizures, in 59% of patients (Varatharaj et al., 2020). Twenty percent of patients are admitted to the intensive care unit (ICU) due to neurological impairment and acute cerebrovascular disease. Once the endotracheal intubation device is removed, they face more serious persistent neurological complications and long-term sequelae (Chen T. et al., 2020; Cipriani et al., 2020).

### Coronavirus Disease-19-Related Encephalopathy and the Application of Imaging Techniques

Based on the diversity of imaging research methods, it provides guidance for us to explore the mechanism of neurological symptoms of COVID-19. The first cases of COVID-19 right encephalitis and ventriculoencephalitis were reported in February 2020. One week after infection, T2 Fluid-Attenuated Inversion Recovery (FLAIR) showed hyperintensity in the right medial temporal lobe and hippocampus, with slight atrophy in the hippocampus, and no abnormal contrast enhancement (Moriguchi et al., 2020). Meanwhile, the first case of acute necrotizing hemorrhagic encephalopathy of COVID-19 was reported in March 2020. Three days after infection, T2 FLAIR showed hyperintensity in bilateral temporal lobes and thalamus with borderline hemorrhagic lesions (Poyiadji et al., 2020).

At the same time, in a study of 253 severely ill patients with acute infection, 10 of 27 (37%) with neurological symptoms showed abnormal signals in the cortex and deep white matter on FLAIR MRI (Kandemirli et al., 2020). In a separate study of 214 patients who tested positive for COVID-19 nucleic acid, diffusion-weighted imaging (DWI) and FLAIR MRI in 2 patients with encephalitis showed mild atrophy of the hippocampus and high signal expression in the right temporal lobe and hippocampus, all confirm inflammation associated with brain parenchyma (Ellul et al., 2020; Mahammedi et al., 2020). From the above study, it can be seen that the development of cerebral neuropathy in patients with COVID-19 is individual and different.

As is known to all, [^18^F]fluorodeoxyglucose positron emission tomography (^18^F-FDG-PET) is widely used in clinical infectious diseases and brain nerve-related diseases. Brain local glucose metabolism can be used to evaluate the indicators of brain nerve activity (Dressing et al., 2021). A study using ^18^F-FDG-PET to assess COVID-19-related encephalopathy found that seven patients showed a low metabolic pattern in the insula and caudate nuclei of the anterior cingulate gyrus of the prefrontal cortex, combined with persistent cognitive and affective dysfunction in the patients (Kas et al., 2021). It is speculated that the disorder of neuronal oxygen metabolism caused by neurotropic SARS-CoV-2 may be related to this disorder (Rodríguez-Alfonso et al., 2021). Therefore, ^18^F-FDG-PET can be used as an important imaging method to detect whether brain metabolism is directly related to SARS-CoV-2 infection in patients with COVID-19 (Rudroff et al., 2021).

Due to the invasion of the nervous system by the SARS-CoV-2 virus, one-half of the total three-fourths of patients with COVID-19 had focal neurological dysfunction. The MRI of 68% of COVID-19 positive patients showed bilateral olfactory bulb edema and T2/FLAIR hyperintensity in the olfactory bulb and olfactory tract. Although olfactory epithelial cells are regenerative cells, the rate of recovery of olfactory function in patients with post-acute sequelae of SARS-CoV-2 infection (PASC) is unclear, and the severity of the patients and the resulting chronic olfactory dysfunction remain unknown (Strauss et al., 2020; Xydakis et al., 2021).

At present, COVID-19 is an independent risk factor for acute ischemic stroke and the onset of ischemic stroke symptoms in the acute stage of infection tends to be in younger adults (Oxley et al., 2020). Assessment of the neuroimaging findings of children following COVID-19 infection found that 74% of COVID-19 positive children with immune-mediated acute disseminated encephalomyelitis showed T2/FLAIR hyperintensity fusion of gray and white matter, and 32% had cranial, spinal, and cauda equina nerve enhancement (Lindan et al., 2021). The manifestations of neurological damage caused by COVID-19 in adults and children are not only very different but also patients gradually tend to be younger. Therefore, it is urgent for us to study COVID-19 encephalopathy.

### Pathophysiology

#### Invasion of the Nervous System by Severe Acute Respiratory Syndrome Coronavirus-2

The SARS-Cov-2 virus is a perineural invasion. The spike (S1) protein of the virus binds to angiotensin-converting enzyme 2 (ACE2) receptors on nasal respiratory cells and olfactory cells and spreads along with the CNS through the nerve-mucosal interface in olfactory mucosa into olfactory and sensory nerves (Baig et al., 2020; Meinhardt et al., 2021). The ACE2 receptor, as the functional receptor and host receptor of the coronavirus, exists on vascular endothelial cells and smooth muscle cells in all organs of the body, spreading widely in the kidney, liver, small intestine, heart, blood vessels, and the brain endothelium (Bourgonje et al., 2020). After entering the blood-brain barrier (BBB), SARS-COV-2 virus binds to the ACE2 receptor of vascular endothelium, inhibits angiotensin 1–7 [Ang-(1–7)] of vascular smooth muscle, induces an oxidative stress response, aggregation of inflammatory factors, and apoptosis of vascular endothelial cells, leading to cerebrovascular endothelial dysfunction, vascular leakage, and activation of immune response (Wang et al., 2016; Li et al., 2020; Varga et al., 2020). Therefore, through ACE2/Ang-(1–7)/mas signaling pathway, the release of brain-derived neurotrophic factor (BDNF) is inhibited, which interferes with glial cells and peripheral cells of neuronal cells and induces the occurrence of mental diseases such as depression, anxiety, and cognitive impairment (Zheng et al., 2014; Kumar et al., 2020).

The renin-angiotensin system (RAS) is closely related to white matter lesions. Once SARS-COV-2 binds to ACE2 receptor overexpression in hypertension and ischemic cerebrovascular lesions, it causes damage to white matter and induces cognitive dysfunction in patients (Sierra et al., 2002). It has been speculated that RAS-targeting drugs can mediate the ACE2/Ang-(1–7)/mas signaling pathway, moderate the binding of ACE2 to the spike protein of the viral particle, and thus play an anti-inflammatory and antivirus role (Pucci et al., 2021).

#### The Cytokine Storm in Coronavirus Disease-19

Recent reports suggest that the binding of SARS-CoV-2 to the nicotinic acetylcholine receptor (nAChR) inhibits parasympathetic (PSNS) and sympathetic (SNS) overstimulation in autonomic dysfunction (DNS) mediated by the COVID-19 syndrome. The resulting severe imbalance of the SNS/PSNS axis promotes cytokine storms (Al-Kuraishy et al., 2021). Biomarkers of the receptor-interacting serine/threonine-protein kinase 1 (RIPK1) activity have been identified in lung pathology samples from patients with COVID-19. The NSP12 gene in SARS-CoV-2 encodes RNA polymerase, which is a highly conserved central component of coronavirus replication and transcription. There is a protein-protein interaction between NSP12 and RIPK1 in the virus, and the activated RIPK1 can promote the expression of viral receptors ACE2 and epidermal growth factor receptor (EGFR). The increase in viral load triggers the release of pro-inflammatory cytokines interleukin (IL)-6 and tumor necrosis factor (TNF), which trigger cytokine storms throughout the body (Xu et al., 2021). RIPK inhibitors have been shown to improve neurodegeneration and inflammatory disease in Alzheimer’s disease, and therefore, RIPK1 could be a potential therapeutic target for COVID-19-related encephalopathy (Mifflin et al., 2020). On the other hand, the main protease (M*^pro^*) of SARS-CoV-2 acts on the host protein nuclear factor (NF)-kB essential modulator (NEMO) of cerebral microvascular endothelial cells and prevents the production of antiviral type I interferon, thereby inducing the apoptosis of microvascular endothelial cells (Wenzel et al., 2021). Once the rupture of capillary endothelial causes cerebral hemorrhage it becomes the most serious sequelae of COVID-19 infection (Reynolds and Mahajan, 2021). It can be concluded that patients with COVID-19 are at high risk of stroke in the context of systemic cytokine storm, hypercoagulability of blood, and long-term endothelial dysfunction mediated by ischemia and hypoxia (Yaghi et al., 2020).

### Severe Acute Respiratory Syndrome Coronavirus-2 Variants

There are adaptive mutations in the SARS-CoV-2 virus. SARS-CoV-2 has developed several variants of concern (VOCs), such as B.1.1.7 (Alpha), B.1.351 (Beta), B.1.617/B.1.617.2 (Delta), and P.1 (Gamma). The emerging variants in the receptor-binding domain (RBD) or N-terminal domain (NTD) of spike glycoprotein in these variants lead to changes in binding with the host ACE2 receptor protein, which results in the formation of high-affinity virus strains that generate immune invasion and vaccine escape in patients, increasing the transmission and infection rates of SARS-CoV-2 (Thye et al., 2021). Among them, B.1.1.7 (P681H) and B.1.617.2 (P681R) variants of SARS-CoV-2 cross the BBB and infect the nervous system by binding neuropilin-1 (NRP-1) on neurons and astrocytes (Chakravarty et al., 2021). NRP-1 is a receptor protein widely existing on the surface of the cell membrane, which promotes the virulence and infectivity of the SARS-CoV-2 virus by binding to the C-end rule (CendR) motif in S1. As a ligand protein of signal transduction, NRP-1 combines with the vascular endothelial growth factor (VEGF), transforming growth factor-beta (TGF-β), and nerve plexins to participate in signal transduction of vascular endothelial inflammation and neuroinflammation, suggesting that NRP-1 can be a protein therapeutic target for COVID-19 encephalopathy (Mayi et al., 2021).

Severe acute respiratory syndrome coronavirus 2 virus and its VOCs are involved in all organs of the body, causing severe circulatory system failure, diffuse cerebral dysfunction, cerebral microhemorrhage, and ischemic hypoxic leukoencephalopathy due to continuous hypoxia in patients (Miners et al., 2020). In addition to improving patients’ respiratory failure, more attention should be paid to the comorbidities of nervous system diseases, mental cognitive changes, and chronic myopathy (Ermis et al., 2021). There is a relative pathogenic relationship between brain injury and lung injury. Patients with severe acute brain injury usually experience respiratory failure, which is undoubtedly a revelation as to whether the SARS-CoV-2 virus directly affects the nervous system or whether there are non-specific complications of COVID-19 systemic disease. Generally, brain imaging changes caused by toxic or acquired metabolic encephalopathy can be used to judge the occurrence and prognosis of brain diseases and to explore the pathogenesis of COVID-19 encephalopathy (Ziaka and Exadaktylos, 2021).

**FIGURE 1:**
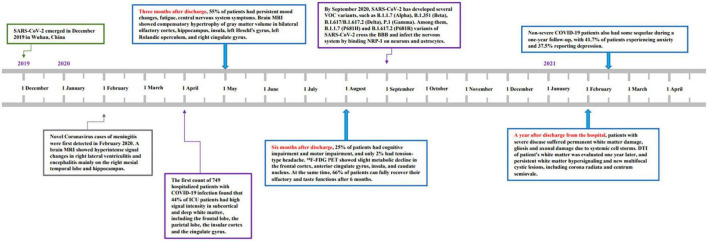
Graph’s x-axis (dates from December 2019 to December 2021) shows the clinical timeline and dynamics of microstructural changes in Coronavirus Disease-19 (COVID-19) encephalopathy in China. Novel Coronavirus cases of meningitis were first detected in February 2020. By September 2020, severe acute respiratory syndrome coronavirus-2 (SARS-CoV-2) had developed several variants of concern (VOC), such as B.1.1.7 (Alpha), B.1.351 (Beta), B.1.617/B.1.617.2 (Delta), and P.1 (Gamma). Three months after discharge, 55% of patients had persistent mood changes, fatigue, central nervous system symptoms. Six months after discharge, 25% of patients had cognitive impairment and motor impairment, and only 2% had tension-type headache. At the same time, 66% of patients fully recovered their olfactory and taste functions after 6 months. A year after discharge from the hospital, 2,433 patients who were discharged from Wuhan hospital were followed up and observed. It was reported that severe patients still had fatigue, sweating, anxiety, and joint pain after review.

This review is aimed to collect, analyze, and summarize the follow-up results of patients with mild and severe disease to date and persistent neurological damage and radiographic changes in patients who have recovered from COVID-19. From December 2019 to December 2021, recovery of the nervous system microstructure of COVID-19 encephalopathy is shown in Figure 1. The longitudinal assessment of neurological microstructure recovery over 3 months, 6 months, and 1 year is shown in Table 1 to further understand the cerebral nerve damage caused by COVID-19 and guide the recovery of COVID-19-related encephalopathy.

**TABLE 1:** Changes in brain microstructure at 3 months, 6 months, and 1 year after rehabilitation.

Duration of symptoms	Symptoms	Imaging technology	Brain imaging features of patients	References
Three months	CRDL	Brain MRI	Extension confluent or multifocal white matter lesions, microhemorrhages, diffusion restriction, and enhancement.	Freeman et al., 2021
Four months	Multiple white matter lesions of brain	Brain MRI	White matter lesions in areas of the brain such as the gray matter junction, the frontal and parietal lobes.	Hellgren et al., 2021
Three months	Focal cerebral microvasculitis	Brain MRI	Focal areas of high signal in the periventricular and subcortical white matter.	Nuzzo et al., 2021
Three months	Olfactory and gustatory dysfunction	Structural MRI, DTI	The bilateral olfactory cortex, hippocampus, insula, left Heschl’s gyrus, left Rolandic operculum and right cingulate gyrus showed increased volume.	Lu et al., 2020
Three months	CRDL	Brain MRI	Ischemic lesions in the parietal and left occipital sites with limited DWI.	Colonna et al., 2020
Three months	Olfactory dysfunction	BOLD-fMRI	Signal was enhanced in the right piriform cortex and the right uncus of the anterior cingulate gyrus.	Ismail and Gad, 2021
Three months	Olfactory function and cognitive impairment	^18^F-FDG-PET	Hypometabolism of olfactory gyrus, right amygdala and hippocampus, right thalamus, bilateral pons brainstem, and bilateral cerebellum.	Guedj et al., 2021
Three months	Brain diffuses dysfunction	Brain MRI, DTI	Left insula lobe, left hippocampus, and left superior temporal gyrus of cortical thickness decreased.	Qin et al., 2021
Six months	Frontal lobe syndrome, emotional disturbances, and deregulation of respiratory failure perception	^18^F-FDG-PET	Persistent low metabolism in the prefrontal cortex, anterior cingulate gyrus, insula, hippocampus, and caudate nucleus.	Kas et al., 2021
Six months	Subjective neurocognitive dysfunction	^18^F-FDG-PET	A few patients had hypometabolic prefrontal cortex, and no significant pathological changes were observed.	Dressing et al., 2021
Seven months	COVID-19-related leukoencephalopathy	Brain MRI, DWI	Cystic leukoencephalomalacia with persistent white matter hypersignal and new multifocal cystic lesions, including corona radiata and centrum semiovale.	Lang et al., 2021
Nine months	Olfactory disorder	Brain MRI	The olfactory cortex, olfactory bulb and sulcus showed no pathological signs.	Cecchini et al., 2022
One year	Visual field loss, blurred vision, and hallucinations	Brain MRI	At 3 months follow-up, brain MRI showed hyperintensity of T2/FLAIR in white matter in the parietal and occipital cortex, with visual impairment. A year later, signs and symptoms persist.	Hixon et al., 2021

## Brain Imaging Findings at 3 Months of Rehabilitation

After 3-month evaluation of rehabilitation, 20% of patients developed fever, 60% cough, 43% increased expectoration, 62% active chest tightness and palpitations, and 60% general fatigue. Among them, 82% of patients had returned to normal after high-resolution computed tomography (HRCT) lung re-examination. Most patients had persistent damage after infection, which may have been caused by extrapulmonary factors (Liang et al., 2020). A recent follow-up of 804 patients who had recovered from COVID-19 infection in two Italian hospitals found that 2 patients were readmitted to the hospital due to acute ischemic stroke without any signs of infection recurrence, which is reasonable to conclude that it may have induced cerebrovascular disease after COVID-19 infection (Mumoli et al., 2020).

A review of the brain MRI of 59 patients with COVID-19 revealed abnormal T2 signaling and limited diffusion in white matter, suggesting that infection-induced COVID-19 is associated with disseminated leukoencephalopathy (CRDL) (Freeman et al., 2021). Brain MRI imaging changes were assessed to observe the relationship between neurocognitive status and leukoencephalopathy 4 months after discharge. Twenty-five patients (71%) were found to have white matter lesions in subcortical frontal and parietal lobes on imaging, and 16 patients (46%) were associated with symptoms of memory delay and cognitive impairment. The incidence of white matter lesions generally increases with age, and COVID-19 associated white matter was seen in severe or elderly patients presenting with chronic impairment and persistent cognitive negative effects (Hellgren et al., 2021). In a follow-up study of critically ill patients with concurrent delirium, imaging in the acute phase showed T2/FLAIR hyperintensity in the occipital and frontal lobes, microbleeds in the corpus callosum, and pia augmentation. After discharge, a standardized icon called Family Confusion Assessment Method (FAM-CAM) was used to assess the patient’s representation cognitive function. The incidence of delirium was decreased from 73 to 24% after 2 months of recovery, suggesting that a neuroinflammatory response to COVID-19 triggers delayed manifestations of delirium and cognitive impairment (Ragheb et al., 2021).

Many studies have reported that neuroinflammation-induced anxiety, depression, and emotional loss of control are prominent in the anterior cingulate cortex (ACC), prefrontal cortex, medial orbitofrontal cortex, and insula (Han and Ham, 2021). A patient who had a negative SARS-CoV-2 reverse transcription PCR test result still had chronic fatigue, fingertip paresthesia, and severe depression 3 months after discharge from the hospital. MRI of the brain was performed and showed focal areas of high signal in the periventricular and subcortical white matter. It was speculated that this had been caused by focal cerebral microvasculitis in the acute phase (Nuzzo et al., 2021). Three months after discharge, patients had persistent mood changes, fatigue, and CNS symptoms, and brain MRI and diffusion tensor imaging (DTI) indicators were evaluated. Compensatory hypertrophy was found in the gray matter volume of the bilateral olfactory cortex, hippocampus, insula, left Heschl’s gyrus, left Rolandic operculum, and right cingulate gyrus. Because of the innate perceptual advantage of the right olfactory nerve, the diffusion of white matter on the right side of the brain is more pronounced than on the left. Compared with the healthy control group, the diffusion parameters, mean diffusivity (MD), axial diffusivity (AD), and radial diffusivity (RD), of the corona radiate (CR), external capsule (EC), and superior frontal-occipital fasciculus (SFF) in the rehabilitation group were significantly lower (*p* < 0.05), while the fractional anisotropy (FA) values were higher, indicating that the cytokines storm induced by the novel coronavirus had sustained damage to the marginal system. The neurologic symptoms of anosmia and memory loss are closely linked to the damage of the hippocampus and cingulate gyrus (Lu et al., 2020). Generally, the hippocampus-prefrontal cortex (HPC-PFC) is involved in the regulation of normal emotions and cognitive functions, anxiety, and depression behaviors, which are directly transmitted through the HPC-PFC pathway. Once the disruption or damage of the pathway occurs, severe depression, anxiety, Alzheimer’s disease, and schizophrenia will be induced (Ruggiero et al., 2021).

A patient with COVID-19-related diffuse leukoencephalopathy appeared in a state of deep coma, the brain MRI showed subcortical hemorrhages, basal ganglia, and cerebellum hemorrhage, with focal high T2/FLAIR signals in the white matter area. However, the critically ill patient regained consciousness and walked independently 3 months later. It can be seen that early intervention treatment of patients with leukodystrophy or cerebral microhemorrhage can achieve the effect of improving prognosis (Witvoet et al., 2021). Another patient was diagnosed with Guillain-Barre syndrome (GBS) due to virus invasion of the peripheral nervous system and sluggish paralysis of limbs. At the time, brain MRI examination revealed a rare microhemorrhage in the white matter of cerebral lobes, albuminocytologic dissociation in cerebrospinal fluid (CSF), and sluggish movement of muscles in limbs. After receiving antiviral, immunoglobulin therapy, and rehabilitation therapy, the patient returned to normal walking 2 months later, which demonstrates that the principle of early detection and early treatment should be followed for COVID-19-related encephalopathy (Colonna et al., 2020).

In another case, functional MRI (fMRI) of the brain was performed after the aroma scented intervention of a patient with hyposmia and dysosmia lasting 3 months, which revealed high blood level-dependent (BOLD) in the right piriform cortex and the right hooks of the anterior cingulate gyrus, confirming persistent damage to the primary olfactory region (Ismail and Gad, 2021). Ninety-five patients with hyposmia were followed up 3 months later and assessed with the Hyposmia Rating Scale (HRS) and Brief Smell Identification Test for Chinese (B-SITC). The rate of hyposmia was decreased from 34.1 to 24.4%, indicating that olfactory function gradually returned to normal after 3 months, independent of the degree of COVID-19 infection (Zhu et al., 2021). ^18^F-FDG-PET review of post-COVID-19 patients with persistent olfactory function and cognitive impairment found low metabolism in the olfactory gyrus, right amygdala and hippocampus, right thalamus, bilateral pons brainstem, and bilateral cerebellum. Among them, the metabolic status of the frontal lobe of 7 hypertensive patients significantly increased after being treated by ACE drugs. It is reasonable to speculate that ACE drugs may be associated with the treatment of COVID-19-related encephalopathy, and it has also been confirmed that the SARS-CoV-2 virus may pass through the infection pathway of the olfactory bulb, and the destruction of the hippocampus and amygdala may lead to the occurrence and development of cognitive impairment in patients (Guedj et al., 2021).

To confirm that there is a certain correlation between the brain microstructure changes of the patients with COVID-19 3 months after recovery and the degree of illness in the acute phase of infection, a routine brain MRI was performed 3 months after discharge. Compared with healthy controls, patients with severe left insula lobe, left hippocampus, and left superior temporal gyrus of cortical thickness were decreased, with cluster volume of 1,742, 327, 366 mm^3^ (*p* < 0.05), and inflammatory markers in serum procalcitonin (PCT) and hippocampal volume contraction were negatively correlated. During the acute stage of infection, cytokine storm induced brain stress response, especially in severe patients. Compared with mild patients, the cerebral blood flow (CBF) values of the bilateral superior medial frontal gyrus and left insula were significantly decreased in severe patients (*p* < 0.05). At the same time, XTRACT was used to evaluate the volume, length, and FA of the subcortical white matter tracts. In mild and severe cases, the volume of white matter fibers was decreased in the right anterior thalamic radiation (ATR), left cingulum bundle, dorsal (CBD), right frontal aslant tract (FAT), forceps minor (FMI), left inferior longitudinal fasciculus (ILF), and right ILF. Especially in critically ill patients, plasma PCT, C-reactive protein (CRP), and IL-6 were increased significantly (*p* < 0.05). This indicates that severe patients still have some brain diffuse dysfunction 3 months after recovery, and the changes in the brain microstructure of severe patients are closely related to the CBF of the insula and negatively correlated with the levels of PCT and IL-6 in serum (Qin et al., 2021). Another study also found that microstructural changes in white matter were closely associated with systemic inflammatory responses and concluded that in patients with COVID-19 encephalopathy, the average apparent diffusion coefficient (ADC) in the white matter region of the genu of the corpus callosum, ATR, and EC was increased with local CRP levels, consistent with the delirium associated with the frontal-subcortical syndrome (Rhally et al., 2021).

The SARS-CoV-2 virus activates pathogenic Th1 cells to induce an immune response, producing a large amount of pro-inflammatory factors, IL-6 and TNF-α, further triggering a cytokine storm throughout the body. Measures of inflammatory mediators and neutrophil/lymphocyte ratio may have prognostic potential. Compared with mild and moderate patients, severe survivors not only have significantly higher leukocytes and neutrophils (*p* < 0.05) but also have high levels of IL-2, IL-6, IL-10, and TNF-α. The increase in systemic inflammatory mediators promoted cerebrovascular endothelial injury and increased blood viscosity and accelerated the occurrence of cardiovascular events and the progression of neurodegeneration (Chen G. et al., 2020; Terpos et al., 2020). Since immune-mediated cytokine storm is directly proportional to the severity of the disease, it is of great significance to closely monitor the imaging changes in brain microstructure and the trend of inflammatory factors in patients 3 months after recovery in order to evaluate the recovery of patients with COVID-19.

## Brain Imaging Findings After 6 Months of Rehabilitation

Sequelae from COVID-19 have been reported to last for at least 6 months, with post-intensive care syndrome (PICS) often present in critically ill patients. Persistent fatigue or muscle weakness and difficulty sleeping were reported in 76% of patients, most notably in women, and anxiety or depression was reported in 23% of patients 6 months after recovery. Due to the physiological characteristics of women, women and the severity of the disease were high-risk factors for persistent psychological disorders. Female patients had higher stress levels, anxiety, and depression levels when facing the post-COVID-19 (Xiong et al., 2021). Another study about COVID-19 survivors who were discharged from the hospital for 3 months found that 48.5% of women had unique sequelae of hair loss, which was speculated to be related to the inflammatory response caused by COVID-19 or the patient’s psychological state during the illness (Huang et al., 2021). Recovery in critically ill patients was associated with a significantly increased risk of abnormal lung diffusion function, fatigue or muscle weakness, and anxiety or depression, which threatens the patient’s quality of life and normal cognitive status (De Moraes De Medeiros et al., 2021; Huang et al., 2021).

In fact, one study counted 203 postings from patients 7-month post-COVID-19 symptoms, statistics showed that 3,503 of the 3,762 (93.2%) patients still had varying degrees of psychiatric symptoms, such as fatigue in 2,652 of the 3,762 (70.5%) patients, respiratory problems in 2,242 of the 3,762 (59.6%) patients, and 1,274 of 3,762 (33.9%) patients. However, there is one indicator worthy of our attention. In total, 87% of patients with cognitive impairment showed no pathological signs on brain MRI, indicating that brain symptoms and signs induced by post-COVID-19 are inconsistent, confirming the importance of follow-up observation of brain imaging changes in post-COVID-19 patients (Davis et al., 2021).

After SARS-CoV-2 infection of the CNS, plasma levels of glial fibrillary acidic protein (GFAP) and neurofilament light polypeptide (NFL) were increased. GFAP is considered a specific protein marker of brain nerve injury. Based on the correlation between damaged areas on the plain computed tomography (CT) scan and serum protein GFAP, it is reasonable to infer that systemic inflammation induces nerve damage in white matter (McMahon et al., 2015; DeKosky et al., 2021). At the same time, some studies collected serum markers of CNS injury in patients at the acute stage and 6 months after recovery and evaluated the severity of the disease, which was found to be positively correlated with the concentration of markers of the CNS injury. The plasma levels of GFAP, NFL, and growth differentiation factor 15 (GDF-15) in severely ill patients were significantly increased (*p* < 0.05). After 6 months, the levels of NFL and GFAP in plasma gradually returned to normal as neuroinflammation subsided, and the GDF-15 level gradually disappeared with the decrease in inflammation. Six months later, neuronal axonal injury is at the repair stage, and astrocytes gradually return to a normal level, further confirming that serum biomarkers can be used as one of the prognostics to evaluate COVID-19 (Kanberg et al., 2021). Tension-type headache symptoms in patients with COVID-19 were positively correlated with inflammatory markers, CRP and IL-6. After 6 months, serum IL-6 levels were gradually returned to normal in recovered patients, and the prevalence of tension-type headache was decreased from 38 to 2% (Caronna and Pozo-Rosich, 2021).

Another follow-up study found that the rate of recovery from symptoms of anxiety, depression, post-traumatic stress disorder (PTSD), cognitive impairment, and motor impairment was dropped from 80 to 25% at 6 months following discharge when compared with patients at 3 months. This indicates that with the severity of the disease, the recovery rate of critically ill patients is low, and most of the diseases develop into chronic nervous system lesions (Evans et al., 2021). There were some cases of severely ill patients with acute brain MRI, which showed the presence of infratentorial cerebral microbleeds (CMBs) in the right temporal lobe, and left basal ganglia, that the patients’ memory and cognitive abilities had returned to normal at the subsequent 8 months (Backman et al., 2021). It was speculated that the symptoms of cognitive impairment were related to regional cerebral glucose metabolism in the acute stage of infection. Therefore, ^18^F-FDG-PET examination of patients with cognitive impairment in the subacute stage found that most of them had cortical hypometabolism in the frontal lobe and parietal lobe. The patients were examined again at 6 months, and no significant changes were found in regional cerebral glucose metabolism and only slight impairment in cognitive tests, that patients’ cognitive status gradually returned to normal with the recovery of cortical metabolism 6 months later (Dressing et al., 2021). Previous studies have provided evidence that decreased cerebellar metabolism is accompanied by decreased brain function, which manifests as persistent hyposmia, memory, and cognitive impairment (Mainland et al., 2005; Bodranghien et al., 2016). To observe neuronal evolution of COVID-19-related neurological symptoms, brain ^18^F-FDG-PET tests were performed thrice on the same group of patients with COVID-19 encephalopathy during the acute phase, 1 month and 6 months later. Results demonstrated that one in seven patients had enhanced white matter and T2 FLAIR hyperintensity in the right prefrontal lobe and caudate nucleus. Most of the patients had extensive manifestations of low metabolism in the brain, such as the frontal cortex, anterior cingulate gyrus, insula, and caudate nucleus, and varying degrees of cognitive impairment and mood disorders, indicating that the corresponding neurological functional areas of the patients were impaired. Compared with the acute phase, the decreased metabolism of the cerebral cortex was improved after 1 month, and only a slight decrease in metabolism was found in the olfactory gyrus after 6 months, and the whole brain metabolism returned to normal, but there were still neurological symptoms, such as anxiety, depression, attention deficit, and executive ability (Kas et al., 2021).

Long-term follow-up of COVID-19 patients’ recovery needs to be combined with pathophysiology in order to understand the remission of patients’ symptoms. Recently, prospective studies have reported that olfactory and gustatory disorders of most infected patients are relieved after 2 weeks, and 66% of patients can fully recover their olfactory and taste functions after 6 months, among which parosmia is a specific indicator of infection in patients (Teaima et al., 2021). At the same time, a study found that the sensitivity of female patients to olfactory and gustatory dysfunction made them vulnerable to SARS-CoV-2. After 7 months, 20% of patients still had parosmia and olfactory disorders (Bussière et al., 2021). The expression of ACE2 in olfactory nerves of a patient with anosmia, olfactory abnormalities, and taste disorders was detected for 15 months. The epithelial marker pan-cytokeratin (PCK) antibody was found to be a strong positive signal, confirming the invasion of the SARS-CoV-2 virus into the CNS. Nine months later, the brain MRI showed no significant structural changes or high signal characteristics in olfactory blubs and cortex. It was reasonable to speculate that the olfactory bulb lesions of the patient had recovered 9 months later (Cecchini et al., 2021, 2022). In previous studies, the RAS system was shown to mediate Ang-(1–7) in the amygdala, hippocampus, and prefrontal cortex, leading to the high expression of ACE2 and thus inducing mood and mental diseases. ACE inhibitors can, therefore, effectively reduce inflammatory mediators, such as IL-6 and CRP, and play a role in improving depression, anxiety, and other adverse emotions (Dagenais and Jamali, 2005; Rocha et al., 2021; Sanches and Teixeira, 2021).

## Brain Imaging Findings at 1 Year of Rehabilitation

At 3 months, a patient’s nervous system should be in gradual recovery, so studies after a year are crucial. To explore the recovery of patients 1 year later, 2,433 patients discharged from Wuhan hospital were followed up and observed. It was reported that critically ill patients still experienced fatigue, sweating, anxiety, and joint pain after review, among which cerebrovascular disease was a high-risk factor for chronic nervous system injury (Zhang et al., 2021). Another study found that patients with non-severe COVID-19 also had some sequelae during a 1-year follow-up, with 41.7% of patients experiencing anxiety and 37.5% reporting depression. Mental state of patients was improved as they recovered from the disease, with the decrease in cortisol levels, patients’ anxiety, and depression gradually improved (Zhou et al., 2021; Zhang et al., 2022). According to preliminary results, patients were recovered after 1 year, especially when comparing the images of patients with patients with mild and severe COVID-19. The longitudinal comprehensive evaluation of abnormal changes in brain imaging during the rehabilitation of patients with COVID-19 was conducted in order to understand whether changes in the brain microstructure persist, to help clinicians appreciate the potential neurological damage of COVID-19, and to provide certain data support for clinical intervention of neurological damage of patients after recovery.

In essence, we should focus our attention on the rehabilitation of white matter disease, which plays an absolute role in regulating cognition and behavior. Aging and loss of white matter integrity can lead to cognitive impairment, and the return of DTI of subcortical white matter to normal levels can be regarded as one of the indicators of patient recovery from a pathological point of view (Bennett and Madden, 2014; Filley and Fields, 2016). Changes in the chronic nervous system after white matter injury were observed in patients with COVID-19. Acute brain MRI showed limited diffusion of white matter in the bilateral deep brain, and axial FA indicated focal destruction of white matter tracts. After 7 months, the patients still had speech impairment and motor function limitation, and a re-examination of brain MRI showed cystic leukoencephalomalacia with persistent white matter hypersignal and new multifocal cystic lesions, such as CR and centrum semiovale. It was speculated that severe patients with COVID-19 suffer from permanent white matter injury, gliosis, and axon injury due to systemic hypoxia and invasion of immune mediators, resulting in chronic neurological sequelae (Lang et al., 2021). Another study also reported symmetrical signs of brain lesions in patients with COVID-19, with diffuse bleeding throughout the white matter region and the knee of the corpus callosum. Repeated MRI reconfirmed the occurrence of acute hemorrhagic leukoencephalopathy (Haqiqi et al., 2021). In these cases, brain MRI showed gradual regression of white matter that occupies lesions in the frontoparietal lobe of the brain, which can be used as a radiological method to identify acute hemorrhagic white matter encephalitis at a convalescent stage and to identify acute disseminated encephalomyelitis (Solis et al., 2017).

In addition to the focus on cerebral white matter, we found another kind of clinical imaging disease—posterior reversible encephalopathy syndrome (PRES). PRES is a syndrome of posterior circulation hyperperfusion mediated by both autoimmune response and endothelial dysfunction. Endothelial dysfunction and coagulation dysfunction lead to the destruction of the BBB, which further induces the occurrence of vasogenic brain edema, presenting intracranial hemorrhage, cerebral thrombosis, and cerebral hemorrhage (Fugate and Rabinstein, 2015; Gewirtz et al., 2021). PRES is present in patients with COVID-19 related to encephalopathy. The clinical manifestations are mainly epileptic seizures, headaches, visual impairment, and mental disorders. Imaging reports show white matter edema and limited diffusion function in bilateral parietooccipital lobes (Yeahia et al., 2022). According to PERS reports, the prevalence of COVID-19 is as high as 4%, and more than half of patients with COVID-19 have complications of hypertension. Diabetes and hypertension undoubtedly increase the susceptibility of patients (Teuwen et al., 2020). Once the SARS-CoV-2 virus invades vascular endothelium, it targets and binds to lipid mediators in the Sphk-S1P-S1PRs pathway to promote the release of inflammatory factors and regulate neuroinflammation and further induce cytokine storm throughout the body (Pan et al., 2021). At the same time, SARS-CoV-2 infection can be predicted by detecting immune cell infiltration and inflammatory factors in endothelial cells (Teijaro et al., 2011). In a follow-up study of patients with COVID-19 who were hospitalized for epileptic seizures during acute infection, the brain MRI showed T2 FLAIR hyperintensity in white matter in the parietal and occipital cortex, accompanied by visual impairment. One year later, the cerebral edema of the patients had subsided, but there was still visual field loss, blurred vision, and hallucinations (Hixon et al., 2021). It is reasonable to assume that severe damage to the CNS causes permanent damage to the cerebral cortex, leading to permanent visual impairment.

In addition to focusing on the physical changes in the brain of post-COVID-19 patients, we found that patients with long-term neurological symptoms are prone to psychological disorders. In the latest study of 4,828 patients with PICS, 37.5% had anxiety and 20% had PTSD (Malik et al., 2022). Another study assessed the psychological status of ICU patients in the Netherlands after 1 year and found that 38.3% developed mental health symptoms, indicating that attention to mental health issues must be taken seriously (Heesakkers et al., 2022). We also found an interesting finding that persistent psychological symptoms, such as anxiety, pain, and depression, were not significantly associated with the severity of the patient’s illness during follow-up in both ICU and general ward patients (Garrigues et al., 2020).

## Conclusion

Coronavirus Disease-2019-associated encephalopathy is a systemic immune response or cytokine storm that induces the development of acute viral encephalitis, acute disseminated encephalomyelitis, such as white matter lesions, anosmia, cerebrovascular disease, and psychiatric symptoms (depression, anxiety, PTSD, and pain disorder) (Troyer et al., 2020; Hazzaa, 2021). COVID-19 was first reported on 31 December 2019, and it has been more than a year since the first patients were discharged from the hospital.

Survivors of COVID-19 infection are of immediate concern, especially for long-term health surveillance of critically ill patients (Rogers et al., 2020). It was found that when brain CT or MRI was performed at least 2 weeks after SARS-CoV-2 virus infection, microangiopathy was detected in the patient’s brain (Radmanesh et al., 2020). In particular, ^18^F-FDG-PET was used to closely monitor the progress of post-COVID-19. Currently, in the 1-year follow-up report, decreased brain metabolism was mostly found in the frontal lobe region (Rudroff et al., 2021). In the future (Chen T. et al., 2020) ^18^F-FDG-PET will be encouraged to be used in clinical follow-up for lifelong observation of patients, which will not only help to detect metabolism abnormalities in multiple organs of the body but also reveal the pathophysiological mechanism of post-COVID-19 at the molecular level. Early monitoring and prognostic adjustment of patients with COVID-19-related encephalopathy are of critical importance. In the acute stage of infection, the use of imaging and close detection of inflammatory factors, timely intervention of patients with neuropsychiatric sequelae, can greatly improve the prognosis of patients (Mazza et al., 2020). Survivors face not only the chronic sequelae caused by the disease but also the psychological pressure brought by social and economic factors. Facing changes in lifestyle brings certain challenges to patients, society, and the world (Denehy and Puthucheary, 2021). Currently, the main clinical treatment for COVID-19 is vaccination and antiviral drug therapy (Luo et al., 2020; Polack et al., 2020; Yin et al., 2022).

Through our review of the symptoms of post-COVID-19, we found that the microscopic changes in the brain of mildest patients could be relieved after 6 months, while ICU patients were prone to long-term sequelae. We believe that due to the diversity and specificity of imaging developments that can reveal the pathogenesis of post-COVID-19 at the micro-level, our report may alert neurosurgeons and radiologists to possible neurological disorders in post-COVID-19 patients. This is different from common neurological encephalopathy, which provides a certain therapeutic reference for doctors’ diagnosis and treatment and patients’ prognosis.

## Data Availability Statement

The original contributions presented in the study are included in the article/supplementary material, further inquiries can be directed to the corresponding author/s.

## Author Contributions

All authors contributed to the manuscript presented methodology, conceptualization, data analysis, and manuscript writing.

## Conflict of Interest

The authors declare that the research was conducted in the absence of any commercial or financial relationships that could be construed as a potential conflict of interest.

## Publisher’s Note

All claims expressed in this article are solely those of the authors and do not necessarily represent those of their affiliated organizations, or those of the publisher, the editors and the reviewers. Any product that may be evaluated in this article, or claim that may be made by its manufacturer, is not guaranteed or endorsed by the publisher.
